# Brain organoids as models of extracellular vesicle–mediated human neural communication

**DOI:** 10.4103/NRR.NRR-D-25-01806

**Published:** 2026-01-27

**Authors:** Giuliana La Rosa, Erika Pascale, Maria Roberta Iazzetta, Edoardo Sozzi, Annalisa Fico, Elvira Immacolata Parrotta, Alessandro Fiorenzano

**Affiliations:** 1 Department of Molecular Medicine and Medical Biotechnology, University of Naples “Federico II,” Naples, Italy; 2 Stem cell laboratory, Department of Medical and Surgical Sciences, University “Magna Graecia,” Catanzaro, Italy; 3 Department of Precision Medicine, University of Campania Luigi Vanvitelli, Naples, Italy; 4 Department of Biosystems Science and Engineering, ETH Zurich, Basel, Switzerland; 5 Stem Cell Fate Laboratory, Institute of Genetics and Biophysics ‘‘A. Buzzati-Traverso,’’ CNR, Naples, Italy; 6 Department of Experimental Medical Science, Developmental and Regenerative Neurobiology, Wallenberg Neuroscience Center, Lund Stem Cell Center, Lund University, Lund, Sweden

**Keywords:** Alzheimer’s disease, amyloid-β, brain organoid, cell–cell communication, extracellular vesicle, neurodegeneration, neurodevelopment, Parkinson’s disease, pluripotent stem cell, α-synuclein

## Abstract

Cellular communication orchestrates human brain development through complex interactions involving adhesion molecules, signaling ligands, extracellular matrix, and extracellular vesicles. While intrinsic genetic programs governing neural differentiation are well characterized, the roles of extrinsic, non-cell-autonomous signaling, particularly extracellular-mediated communication, remain poorly understood. Here, we review recent advances in three-dimensional brain organoids derived from human pluripotent stem cells as physiologically relevant models that recapitulate key aspects of human neurodevelopment, enabling detailed study of extracellular vesicle-mediated intracellular signaling. We highlight how organoid systems facilitate the investigation of extracellular vesicle cargo dynamics and their influence on neural cell fate, migration, and circuit assembly, as well as their involvement in neurodegenerative disorders, such as Alzheimer’s and Parkinson’s diseases. These insights show the potential of brain organoids to unravel complex cellular interactions and inform biomarkers discovery and therapeutic strategies for neurological diseases.

**Facts**

• Brain organoids provide a human three-dimensional model to study extracellular vesicle (EV)–mediated communication, overcoming animal/two-dimensional model limitations.

• EVs actively regulate neural development and homeostasis by transferring bioactive molecules that influence differentiation and plasticity.

• Organoid studies show EV cargo is dynamically regulated by cell type, stage, and context, marking them as precise signaling entities.

• In neurodegenerative diseases, EVs propagate pathogenic proteins and serve as accessible sources of biomarkers.

**Open questions**

• Are EVs active drivers or passive signals in neural development and neurodegeneration?

• What mechanisms control EV cargo sorting in neural cells across states?

• How is EV signaling controlled in space and time within three-dimensional brain tissues?

• How can we trace EV origin and target cells in complex systems?

• Can standardized, quantitative EV assays be developed for organoids?

## Introduction

**Figure 1:**
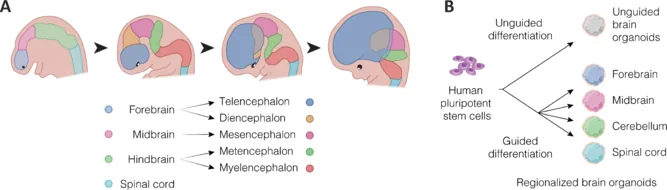
Human neurodevelopment and region-specific organoid model. (A) Schematic representation of human brain development. (B) Generation of region-specific brain and spinal cord organoids from human pluripotent stem cells.

Human brain development is governed by a complex interplay between intrinsic genetic programs and extrinsic signaling cues (Breau et al., 2017; Zhou et al., 2024), which together ensure the formation of a functional nervous system (**Figure 1A**). While significant advances have clarified the roles of intrinsic regulators, such as transcriptional networks and epigenetic modifications that drive neural induction and lineage commitment, our understanding of extrinsic, non-cell-autonomous signaling (e.g., signals originating from neighboring or distinct cells rather than from with the cell itself) remains limited (Breau et al., 2017; Krohn et al., 2023; Coppola et al., 2025).

Within this dynamic developmental environment, cell-cell communication is essential for coordinating neurogenesis, cell migration, and axon guidance. These interactions are mediated by a broad repertoire of cues, including secreted factors, adhesion molecules, components of the extracellular matrix (ECM), and extracellular vesicles (EVs) (Marin et al., 2000; Stiles and Jernigan, 2010). Such extracellular signals play pivotal roles in regulating cell numbers, specifying the identities of neural cells, and directing the spatial organization and functional integration of emerging neural circuits. Among these extrinsic mediators, EVs have recently gained attention as promising, yet still underexplored, contributors to brain homeostasis and disease (Bahram Sangani et al., 2021).

EVs are membrane-bound nanoparticles secreted by virtually all cell types, forming a heterogeneous population that transports diverse bioactive cargo, including DNA, mRNA, proteins, and small non-coding RNAs such as microRNAs (miRNAs) (Kumar et al., 2024). By transferring this molecular content to neighboring or distant cells, EVs mediate horizontal communication that influences the behavior of recipient cells independently of direct contact. Increasing evidence highlights EVs as key regulators of developmental processes, including brain patterning, neurogenesis, and tissue homeostasis (Filannino et al., 2024). Importantly, EVs can travel both locally and over long distances, facilitating signaling between diverse neural populations and thereby contributing to the complex cellular cross-talk that drives brain development (Oliveira et al., 2019; Berto et al., 2025).

Despite their abundance and emerging importance, the precise functional roles of EVs in brain development, particularly in critical processes such as cell fate specification, migration, and neural circuit assembly, remain poorly understood and warrant deeper investigation (Bahram Sangani et al., 2021; **Figure 1A** and **B**). Increasing evidence implicates dysfunctional EV signaling in the pathogenesis of both neurodevelopmental and neurodegenerative disorders. To date, EV research has predominantly focused on their use as diagnostic biomarkers in biofluids, such as blood, urine, and cerebrospinal fluid (Xu et al., 2025). For example, proteins carried by urinary EVs have been shown to predict neurodegenerative diseases, including multiple sclerosis, Parkinson’s disease (PD), and Huntington’s disease (Li et al., 2023c), while specific miRNA profiles in blood-derived EVs correlate with Alzheimer’s disease (AD) progression (Aharon et al., 2020). Moreover, altered levels of brain-derived plasma EVs have been linked to cognitive impairments, showing their diagnostic potential. Nonetheless, beyond these biomarker applications, the roles of EVs in orchestrating intercellular communication and regulating neural development and function remain largely unexplored (Oliveira et al., 2019; Berto et al., 2025).

Crucially, the identity, cellular origin, molecular composition, and precise functional contributions of EVs within the human central nervous system (CNS), especially during early brain development and neurodegenerative processes, are still insufficiently characterized. This knowledge gap is partly due to technical challenges associated with studying human brain tissue, including limited accessibility, difficulties in genetic manipulation, and constraints on scalability (Pascale et al., 2020; Fiorenzano et al., 2021a; Kajtez et al., 2021).

Although rodent models have yielded valuable insights, their translational relevance is limited by fundamental species-specific differences in brain structure and developmental timelines (Dawson et al., 2010; Fisher and Bannerman, 2019). Additionally, isolating and studying EVs directly from rodent brains *in vivo* is complicated by low vesicle yield, cellular heterogeneity, and the inability to track EV release and dynamics in real time (Huang et al., 2023; Shi et al., 2023). These challenges have significantly hindered our ability to study the role of EVs in fundamental biological processes such as cell–cell communication, spatial organization, and progenitor cell regulation within the developing human brain. The shortcomings of traditional animal models are particularly evident when modeling complex, multifactorial disorders, such as AD and PD, where the interplay of genetic, epigenetic, and environmental factors complicates mechanistic understanding (Dawson et al., 2018; Fisher and Bannerman, 2019). This highlights the urgent need for innovative, human-relevant models to elucidate the contributions of EV-mediated signaling in brain development and disease.

To address these challenges, three-dimensional brain organoids derived from human induced pluripotent stem cells (iPSCs) have emerged as a powerful and physiologically relevant platform for modeling human brain development and disease (Lancaster et al., 2013; Fiorenzano et al., 2021c; Kajtez et al., 2025). Advances in stem cell technology now enable unprecedented exploration of both normal neurodevelopmental processes and patient-specific molecular dysfunctions (Fiorenzano et al., 2025). Since tissue function depends on the complex interplay between cellular components and their microenvironment, accurately recapitulating cell behavior *in vitro* requires models that closely mimic the native cellular context. The adventage of iPSC technology offers unique opportunities to investigate the diverse causes of neurodevelopmental factors (Wernig et al., 2008; Soldner et al., 2009; Bose et al., 2022).

Brain organoids faithfully reproduce key features of the developing human brain, including spatial organization, cellular diversity, gene expression patterns, and developmental trajectories (Kanton et al., 2019; Velasco et al., 2019; Sozzi et al., 2022b). As a result, they provide a valuable system for studying both intrinsic genetic programs and extrinsic signaling mechanisms. While brain organoids have been widely employed to study intrinsic pathways involved in neural differentiation, patterning, and lineage commitment, their potential to model EV-mediated intercellular communication is only now beginning to be explored (Reumann et al., 2023; Fiorenzano et al., 2024; Kim et al., 2025b). Importantly, three-dimensional organoid cultures promote enhanced EV secretion compared to traditional two-dimensional (2D) monolayers, likely due to their greater cellular complexity, polarization, and more tissue-like microenvironments (Thippabhotla et al., 2019). This makes organoids particularly well-suited for profiling EV populations and investigating how EV cargo influences neurodevelopmental processes.

In this review, we highlight how brain organoids offer a powerful, human-specific platform to dissect the complex dynamics of cell–cell interactions during brain development. Among the diverse mediators of intercellular communication, we focus on EVs, which have gained increasing attention for their emerging roles in regulating neurodevelopmental processes and their potential diagnostic and therapeutic applications in brain disorders. By studying EV secretion and regulation in brain organoids, and decoding their molecular cargo, researchers can uncover new layers of regulatory control, spanning post-transcriptional gene regulation, protein modifications, and non-cell-autonomous signaling pathways. These insights not only deepen our understanding of human brain development but also pave the way for discovering novel biomarkers and therapeutic strategies relevant to both neurodevelopmental and neurodegenerative diseases.

## Search Strategy

To identify relevant literature for this narrative review, we conducted a search of the PubMed and Web of Science databases for articles published between January 2000 and October 2025. The following search terms were used in various combinations: brain organoid, cerebral organoid, pluripotent stem cell, induced pluripotent stem cell, extracellular vesicle, exosome, neuroexosome, neurodevelopment, neurodegeneration, Alzheimer’s disease, Parkinson’s disease, amyloid-β, α-synuclein, and cell-cell communication.

Titles and abstracts were initially screened for relevance to EV-mediated intercellular communication in human brain organoid models. Full-text articles were subsequently reviewed to ensure they included detailed descriptions of EV characterization, organoid model systems, or functional studies of EV cargo. Only articles published in English were included. Studies focusing on brain organoids and neural EV systems were included. Studies focusing exclusively on EVs derived from non–brain organoid models were excluded.

Additionally, relevant references cited in the selected articles were manually screened to ensure comprehensive coverage. Most of the chosen literature (**~**75% of references) was published between 2020 and 2025, reflecting the rapid recent advances in organoid technology and EV research.

## Brain Organoids as a Model for Human Brain Development

The use of human pluripotent stem cells (hPSCs) has long promised to overcome the limitations of animal models by enabling the recreation of fundamental molecular and architectural features of the human brain (Kadoshima et al., 2013; Lancaster and Knoblich, 2014; Jo et al., 2016). iPSCs, reprogrammed from healthy or diseased individuals, further allow the modeling of neurodevelopmental and neurodegenerative disorders, providing critical insights into how patient-specific genetic backgrounds drive disease phenotypes (Soldner et al., 2009; Laperle et al., 2020; Giacomoni et al., 2024b; Parrotta et al., 2025).

For decades, 2D adherent cultures have served as the primary platform for differentiating hPSCs into specific neuronal subtypes, including glutamatergic, GABAergic and dopaminergic neurons (Nilsson et al., 2021). While 2D cultures offer advantages, such as generating highly homogeneous, pure cell populations and facilitating genome editing, their monolayer format inherently limits the complexity of cell-cell interactions and three-dimensional tissue architecture (Liu et al., 2018). Despite these constraints, 2D systems remain invaluable for disease modelling, cell therapies and other high-throughput applications (Tiklová et al., 2020; Shrigley et al., 2021). The advent of advanced three-dimensional human stem cell models, specifically brain organoids, has transformed the study of the human brain. These organoids recapitulate the intricate cytoarchitecture, cellular diversity, and dynamic intercellular communication of the developing human brain, offering a physiologically relevant platform to investigate neural tissue *in vitro* (Fatehullah et al., 2016; Fiorenzano et al., 2025). This approach has opened new avenues for exploring brain development, disease mechanisms, and cellular communication within a human-specific context that was previously difficult to access. By enabling the study of complex neurobiological processes in a controlled, human-relevant system, brain organoids provide unprecedented opportunities to advance both fundamental research and translational applications (Li et al., 2023b; Birtele et al., 2025). Brain organoid differentiation protocols are generally divided into two categories: unguided (self-patterned) and guided approaches, each with distinct advantages and limitations. Unguided differentiation relies on the intrinsic capacity of hPSCs to endogenously produce morphogenetic and patterning factors, driving self-organization into complex, multicellular structures that spontaneously mimic multiple human brain regions (Lancaster et al., 2013; Quadrato et al., 2017; Johansson et al., 2022). These organoids, often termed cerebral organoids, display heterogeneous cellular compositions that include diverse regional identities and recapitulate key developmental milestones, such as neuroepithelial rosette formation and cortical-like layering (Qian et al., 2019; Da Conceicao et al., 2025). This diversity and complexity make unguided organoids particularly well suited for studying cellular communication and cell–cell interactions during early brain development, as they capture a broad spectrum of neural and non-neural cell types in a dynamic environment. However, this heterogeneity can also be a drawback, as it introduces variability and may generate tissue identities corresponding to distant brain regions, complicating direct comparisons to specific *in vivo* structures (Velasco et al., 2019).

Conversely, guided differentiation protocols apply precise, timed extrinsic cues to steer hPSCs toward defined brain regions, including cortex, striatum, midbrain, cerebellum and more (Qian et al., 2016; Fiorenzano et al., 2021c; Sozzi et al., 2022a; Atamian et al., 2024; **Figure 1B**). This targeted approach improves reproducibility and yields more homogeneous populations, enabling detailed investigations of extrinsic signaling pathways and cell interactions within specific tissue contexts, particularly during later stages of differentiation (Rezaei et al., 2023; Zhang et al., 2023b). Guided organoids thus provide a more controlled platform for dissecting region-specific developmental processes and disease mechanisms. The trade-off is a narrower cellular diversity and reduced ability to model the full complexity of the human brain. Ultimately, unguided and guided brain organoid protocols complement each other, offering synergistic tools for understanding the multifaceted biology of human brain development and disease. Leveraging the strengths of both approaches allows researchers to explore the spectrum of cellular communication from broad, early developmental contexts to refined, tissue-specific processes (Qian et al., 2019; Rizzuti et al., 2024).

## Reconstructing Cellular Interplay with Advanced Brain Organoid Systems

Current state-of-the-art brain organoid systems strive to more accurately recapitulate the intricate cellular interactions that drive human brain development and sustain its complex functions (Oliveira et al., 2019; Birtele et al., 2022; Galimberti et al., 2025). However, one of the fundamental challenges in this attempt to model brain development *in vitro* lies in the diverse developmental origins and temporal trajectories of the brain’s constituent cell types. Neurons, astrocytes, and oligodendrocytes originate from neuroectodermal progenitors but differentiate along distinct lineage pathways and at different developmental stages. Meanwhile, microglia and vascular cells arise from non-neural mesodermal progenitors, following entirely separate ontogenetic programs (Ao et al., 2021; Hu et al., 2023; Liddell et al., 2024; Luongo et al., 2024). This developmental divergence poses a significant obstacle to simultaneously generating all these cell types within a single organoid, as traditional protocols typically favor one lineage or developmental timeframe at the expense of others (Cakir and Park, 2022; Faravelli et al., 2025).

**Figure 2:**
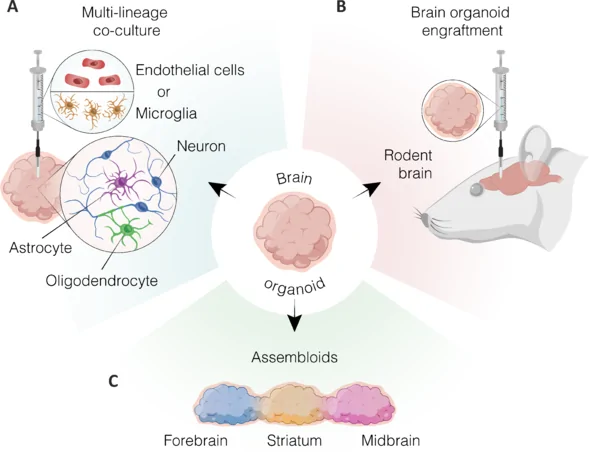
Advanced brain organoid platforms for model human neurobiology *in vitro* and *in vivo*. (A) The central brain organoid represents a dynamic platform that can be further developed to model different aspects of human neurobiology. Fusion of region-specific organoids, such as those derived from the forebrain, hindbrain, and spinal cord, gives rise to complex assembloids that emulate inter-regional communication and network formation *in vitro*. (B) Within the organoid, co-cultures of neurons, astrocytes, oligodendrocytes, microglia, and vascular elements recapitulate the intricate neuroimmune and neurovascular interactions that sustain brain homeostasis. (C) When engrafted into the rodent brain, the organoid undergoes further maturation and vascular integration, promoting neuronal differentiation, gliogenesis, and functional incorporation of human-derived cells into host neural circuits.

The inability to fully capture this cellular heterogeneity within a single three-dimensional structure limits the capacity of brain organoids to model the full spectrum of cell-cell communication and tissue architecture observed in the human brain (Krohn et al., 2023; Santacreu-Vilaseca et al., 2025). Overcoming these challenges is critical for advancing organoid technologies into more human-relevant platforms that can faithfully mimic the cellular complexity and dynamic interplay of human brain regions in both health and disease contexts. To tackle these challenges, innovative approaches have emerged that combine regionally specified organoids or incorporate external cell types, thereby enhancing the fidelity of cellular communication modelling (Pașca et al., 2022; Kajtez et al., 2025; **Figure 2**).

In the following sections, we present and discuss the latest brain organoid technologies designed to model intercellular and extracellular communication within the developing human brain. We focus on systems that more accurately reproduce the cellular diversity, connectivity, and dynamic signaling environment underlying brain development. These organoid-based approaches enable the dissection of extracellular mechanisms that regulate neural patterning, cell–cell interactions, and tissue organization, thereby advancing our understanding of how these processes contribute to both normal development and disease. By recapitulating these complex extracellular interactions *in vitro*, brain organoids provide a powerful experimental platform for uncovering mechanistic principles and informing therapeutic strategies for neurological disorders.

### Co-culture strategies for multicellular brain organoids

Recent advances in brain organoid technology have increasingly employed multicellular co-culture systems to more accurately recapitulate the cellular complexity of the human brain (Guttikonda et al., 2021; Nilsson et al., 2021; Giacomoni et al., 2024a). The addition of external cell populations, such as microglia and astrocytes, has been shown to enhance neuronal maturation, synaptic organization, and immune functionality within organoids (**Figure 2A**). Microglia in particular, introduced either by targeted injection or through co-differentiation protocols, allowed detailed investigation of neuroimmune interactions and synaptic pruning mechanisms (Sabate-Soler et al., 2022; Schafer et al., 2023). These cells are central to maintaining brain homeostasis, continuously monitoring the neural environment and interacting with other cell types. During development, microglia regulate critical processes such as synaptic pruning, neuronal circuit refinement, and immune modulation, whereas in the adult brain, they serve as first responders to injury or infection (Bennett et al., 2021; Cserép et al., 2021; Lin et al., 2021). Advances in iPSC technology have allowed the generation of microglia-like cells that retain key molecular and functional properties of primary human microglia, including phagocytosis and cytokine signaling (Salat and Tolosa, 2013; Bershteyn et al., 2017; Tremblay et al., 2019; Zhang et al., 2023a). Co-culture of these cells with neurons or neural progenitors has provided valuable insights into neuroimmune interactions in both physiological and pathological contexts, including viral infection and neurodegenerative disease. Emerging organoid models also integrate microglia with vascular components, enabling the reconstruction of neuroimmune and neurovascular interactions in a three-dimensional human setting (Cakir et al., 2019; Bhaduri et al., 2020; Shi et al., 2020). These co-culture approaches thus provide a robust platform to investigate microglia–neuron crosstalk, glial maturation, and disease-relevant phenotypes in a context that closely resembles the human brain microenvironment.

Similarly, the inclusion of astrocytes and oligodendrocytes supports neuronal growth and maturation (Marques et al., 2016; Sloan et al., 2018). Beyond their canonical roles in myelin production and axonal insulation, oligodendrocytes provide critical trophic and metabolic support necessary for brain development and function. Human iPSC-derived oligodendrocyte precursor cells in 2D cultures have enabled the study of genotype–phenotype relationships and disease mechanisms (Wang et al., 2013). However, 2D systems lack the structural complexity and cellular diversity required to model processes such as migration, differentiation, myelination, and neuron–glial interactions. Transplantation of human iPSC-derived oligodendrocyte precursor cells into animal models has demonstrated their ability to migrate, differentiate, and remyelinate axons, restoring conduction and function in demyelination or spinal cord injury models (Cao et al., 2010). In cerebral organoids, mature oligodendrocytes are initially rare, with myelinating cells appearing only after prolonged culture. These cells can be incorporated through directed differentiation within the organoid or by introducing pre-differentiated populations, enabling the study of neuron–glia interactions, myelination, and neuroimmune signaling (Lin et al., 1993, 2021; Ferrini et al., 2013). Human iPSC-derived oligodendrocyte precursor cells integrated into organoids can mature into myelinating oligodendrocytes, enhancing axonal insulation and supporting neuronal metabolism, while astrocytes provide trophic support and maintain homeostasis (Birey et al., 2022).

### Brain organoid transplantation

The crosstalk between microglia, vascular cells, and neurons can alternatively be reconstituted through organoid transplantation into rodent brains, a strategy that has recently gained attention (Fiorenzano et al., 2021b; Pașca et al., 2025; **Figure 2B**). Although not a fully humanized model, this approach allows organoids to develop within an *in vivo* environment, where they can receive vascularization and trophic support from host-derived cells. Following transplantation, human brain organoid grafts exhibit progressive neuronal differentiation and maturation, gliogenesis, axonal outgrowth into multiple host brain regions, and infiltration of microglia originating from the rodent brain (Mansour et al., 2018). Remarkably, these *in vivo* grafts display functional integration, engaging host neuronal circuits involved in behavior and motor control (Revah et al., 2022). Beyond enabling the study of cell–cell interactions and long-range connectivity, organoid transplantation provides a powerful system to investigate human neuronal maturation, synaptic integration, and network activity within complex brain circuits. A notable example is the study by Zheng et al. (2023), in which iPSC-derived midbrain organoids were transplanted into the striatum of 6-hydroxydopamine-induced mice. The grafts demonstrated both functional integration into host striatal circuits and partial recovery of motor deficits. While organoid transplantation offers valuable insights into neuronal maturation, axonal wiring, and host–graft interactions, it remains unsuitable for clinical applications due to intrinsic heterogeneity and limited molecular control. Its principal contribution lies in advancing basic neurobiology, particularly the study of dopaminergic neuron development, aging, and stress responses (Umans and Gilad, 2025). For therapeutic purposes, however, 2D stem cell–derived dopaminergic progenitors continue to represent the gold standard, providing precise molecular specification, reproducibility, and stable integration following transplantation, outcomes that organoid grafts have yet to achieve (Piao et al., 2021; Kirkeby et al., 2023; Storm et al., 2024; Sozzi et al., 2025).

### Assembloids for interregional connectivity and circuitry

Conventional cerebral organoids can capture many features of regional development, yet their stochastic patterning limits the study of directed interactions between defined brain areas. To address this limitation, assembloids, engineered by fusing organoids with distinct regional identities, have emerged as a versatile strategy to reconstruct developmental processes and functional circuits *in vitro* (Marton and Pașca, 2020; Levy and Paşca, 2025; **Figure 2C**).

One of the earliest applications involved the fusion of dorsal and ventral forebrain organoids, which enabled modeling of interneuron migration into cortical tissue. Using fluorescent labeling and live imaging, investigators demonstrated C–X–C motif chemokine receptor 4-dependent saltatory migration and subsequent integration of interneurons into cortical circuits (Bagley et al., 2017). When applied to patient-derived iPSCs carrying Timothy syndrome mutations, the system revealed migration abnormalities, showing its utility for probing neurodevelopmental disorders and testing therapeutic strategies (Birey et al., 2022). The same principle has been extended to model descending motor pathways. Cortical-, hindbrain-, and spinal cord–like spheroids have been combined with human skeletal muscle to generate cortico-motor assembloids. Within these systems, corticospinal neurons project to spinal motor neurons, which in turn innervate muscle fibers. Functional validation by rabies tracing, calcium imaging, and patch-clamp recordings confirmed circuit integrity, while optogenetic or chemical stimulation of cortical neurons reliably triggered muscle contraction (Andersen et al., 2020). These assembloids remain viable for extended periods, providing an unprecedented model of human motor circuit formation and neuromuscular disorders.

Other assembloid configurations have been designed to reconstruct higher-order networks. By integrating cortical, striatal, and thalamic organoids in a closed-loop arrangement, investigators have generated cortico-striatal–thalamic–cortical circuits *in vitro*. Functional analyses revealed the emergence of synchronous neuronal activity and verified connectivity across modules. Importantly, assembloids derived from ASH1, such ashistone lysine methyltransferase (ASH1L) mutant lines displayed aberrant network dynamics, offering a human platform for studying circuit dysfunction in neurodevelopmental conditions such as autism spectrum disorder and Tourette syndrome (Miura et al., 2020).

Midbrain-centered assembloids have also provided new opportunities to study dopaminergic systems. Ventral midbrain, striatal, and cortical organoids can be fused into midbrain–striatal–cortical organoids, which exhibit long-range dopaminergic projections to both striatal and cortical tissues (Reumann et al., 2023). These models recapitulate key features of nigrostriatal and mesocorticolimbic pathways, enabling investigation of dopaminergic maturation, selective vulnerability, and drug responses. Notably, chronic cocaine exposure produced persistent structural and functional alterations in midbrain–striatal–cortical organoids, highlighting their potential for modeling addiction and therapeutic interventions. Assembloid systems have also been engineered to incorporate positional information. For instance, the introduction of a fibroblast growth factor 8 expressing organizer into elongated cortical organoids established rostrocaudal gradients, leading to the emergence of region-specific transcriptional programs reminiscent of frontal and temporal cortical identities (Bosone et al., 2024). This polarized architecture provided a tractable model of early arealization and revealed pathogenic effects of FGFR3 mutations associated with cortical malformations and intellectual disability.

## Extracellular Vesicles: Insights into Cellular Communication

EVs are nanoscale membrane-bound structures surrounded by a double lipid membrane and secreted by all types of cells (Kirkeby et al., 2023). Originally considered as by-products of cell turnover, EVs are now recognized as key mediators of both intracellular signaling and intercellular communication, able to transfer proteins, lipids and genetic material both within cells and to nearby or distant target cells (Gurung et al., 2021). Through this transfer of molecular information, EVs orchestrate a wide range of biological processes, from tissue homeostasis to immune modulation (Berumen Sánchez et al., 2021). Importantly, EVs are also implicated in determining cell fate and developmental commitment, where they help shape microenvironments, guiding the differentiation of stem and progenitor cells and influencing organogenesis (Tsai et al., 2022; Al-Sharabi et al., 2024; Kim et al., 2025a). These properties highlight EVs not only as messengers, but also as regulators of crucial decisions in cell development. Alongside this, they play a role in regenerative responses, promoting the repair of damaged tissues, not only in physiological conditions but also in pathological contexts, where they can spread altered signals that contribute to the progression of degenerative or neoplastic diseases (Varderidou-Minasian and Lorenowicz, 2020; Yin et al., 2020; Xia et al., 2022; Guo et al., 2023; Yoo et al., 2025). Hence, EVs are versatile and dynamic mediators, able to combine physiological and pathological functions in different biological contexts. Over the past decade, research on EVs has grown exponentially, thanks to advances in isolation techniques (Welsh et al., 2024), high-resolution imaging, next-generation sequencing, and multi-omics technologies (Roy et al., 2021; Fan and Poetsch, 2023; Zhu et al., 2023; Welsh et al., 2024; Yang et al., 2024). These tools have revealed the remarkable diversity of EV cargo and functions, establishing their involvement in both physiological and pathological contexts, including cancer, immune disorders, and neurodegeneration. However, significant gaps remain. The molecular rules governing selective cargo loading, the precise mechanisms of EV release and uptake, and the context-specific outcomes of EV signaling are not yet fully understood.

### Extracellular vesicles: definition, classification, and molecular features

At present, the term EVs is increasingly used as an umbrella definition that includes all nanometric-sized, membrane-bound particles actively released into the extracellular space, regardless of their biogenesis or size. This nomenclature mirrors a conceptual shift towards recognizing EVs as a heterogeneous continuum rather than as rigidly defined entities. Traditionally, EVs were grouped into three main subtypes: exosomes, microvesicles, and apoptotic bodies. Exosomes (**~**30–150 nm) originate within the endosomal system as intraluminal vesicles of multivesicular bodies and fuse with the plasma membrane to release their contents; microvesicles (**~**100–1000 nm) detach directly from the plasma membrane; apoptotic bodies (> 1000 nm) that are generated during the final stages of programmed cell death (Gurung et al., 2021). In addition to this classic framework, further EV subtypes have been described. This includes: (i) migrasomes, originated from retraction fibers and released during cell migration; (ii) ectosomes, a broad class of EVs generated by external budding of the plasma membrane and including conventional microvesicles and oncosomes, which are substantially larger (**~**1–10 μm) and often released by tumor cells; (iii) secretory autophagosomes, released through unconventional secretion pathways, particularly under specific conditions, such asserum deprivation (van Niel et al., 2022). The identification of these additional vesicle populations highlights the complexity of EV biogenesis pathways and challenges the strict use of subtype-based nomenclature in the absence of clearly defined markers (**Additional Table 1**). Since sizes overlap and no single molecular marker univocally identifies a specific subtype, classification is now based on multiple parameters, such as biogenesis, morphology, load, and cellular origin, rather than size alone. Accordingly, current approaches increasingly rely on the combined assessment of physical properties, molecular characteristics, and functional attributes to describe EV populations in a biologically relevant manner. EVs exhibit remarkable molecular diversity, reflecting the physiological or pathological state of the producing cell. They contain proteins, lipids, metabolites, DNA fragments, messenger RNAs, and various classes of non-coding RNAs (Kumar et al., 2024; Papoutsoglou and Morillon, 2024). It is believed that cargo loading is a selective and regulated process, although the precise mechanisms are not fully defined (Dixson et al., 2023). Markers commonly used for EVs characterization include tetraspanins (CD9, CD63, and CD81), endosomal sorting complex required for transport (ESCRT) associated proteins, such asALG-2-interacting protein X (Alix) and tumor susceptibility gene 101 protein (TSG101) and heat shock proteins (HSP70 and HSP90), along with integrins and other cell type-specific molecules (Gurung et al., 2021; Kirkeby et al., 2023). However, these markers are not exclusive to a single EV subtype, reinforcing the need for multiparametric characterization strategies in defining EV identity. Taken together, these molecular and structural characteristics highlight both the heterogeneity of EVs and the difficulties in their fine classification, reinforcing their central role as multi-tasking mediators of cellular communication.

**Additional Table 1:** An overview of extracellular vesicle subtypes

Subtype	Size	Marker	Biogenesis/release
Exosomes	30-150 nm	Tetraspanins (CD9, CD63 and CD81), ESCRT proteins (Alix, and TSG101), integrins, heat shock proteins (Hsp70, and Hsp90)	Formed as intraluminal vesicles by inward budding of the endosomal membrane via ESCRT-dependent or independent mechanisms or microautophagy; released upon MVE fusion with the plasma membrane
Ectosomes (including microvescicles and oncosomes)	100-10000 nm	Integrins, selectins, CD40 ligand, flotillin-2, ARF6, and phosphatidylserine	Formed by outward budding of the plasma membrane and subsequent scission or pinching off from membrane protrusions
Apoptotic bodies	>1000 nm	Annexin V, and phosphatidylserine	Released from apoptotic cells as a result of plasma membrane blebbing and fragmentation
Migrasomes	500-3000 nm	TSPAN4	Generated from retraction fibres and released during cell migration
Secretory autophagosomes	Not determined	LC3	Secretory autophagosomes form via macroautophagy and are released by plasma membrane fusion

The Table summarizes their typical size ranges, characteristic markers, and biogenesis/release pathways, highlighting key features that distinguish each subtype. Alix: ALG-2-interacting protein X; ARF6: ADP-ribosylation factor 6; CD; cluster of differentiation; ESCRT: endosomal sorting complex required for transport; Hsp: heat shock protein; LC3: microtubule-associated protein 1 light chain 3; MVE: multivesicular endosome; TSPAN4: tetraspanin-4; TSG101: tumor susceptibility gene 101 protein.

In a field marked by extreme heterogeneity and blurred biological boundaries between vesicular populations, the need for shared standards to define, characterize, and interpret EVs has become increasingly urgent. Over the past decade, guidelines developed by the International Society for Extracellular Vesicles have progressively steered the field toward a more rigorous and pragmatic framework, emphasizing integrated physical, molecular, and functional criteria rather than rigid and often experimentally indistinguishable subtype assignments.

Central to this effort are the Minimal Information for Studies of Extracellular Vesicles guidelines, designed to enhance rigor, transparency, and reproducibility in EV research. Recent updates build on the 2018 consensus by strengthening recommendations for EV isolation, characterization, and functional analysis, with a strong emphasis on multiparametric approaches. Key principles include the use of complementary isolation strategies, panels of positive and negative protein markers instead of single-marker identification, and functional assays that discriminate EV-associated activities from effects driven by co-isolated non-vesicular components.

Concomitantly, EV classification is increasingly framed around experimentally accessible parameters, such as biogenesis, molecular composition, and functional behavior, rather than fixed subtype labels that may not be resolvable in complex biological samples. The guidelines further stress the importance of transparent reporting and community data sharing, calling for detailed documentation of culture conditions, biofluids or supplements, normalization strategies for EV dosing (e.g., particle number, protein content, or producer cell number), and systematic assessment of potential non-vesicular contaminants, including lipoproteins and protein aggregates. Recent community-wide evaluations of the EV literature show both the progress made and the challenges that remain, revealing substantial variability in adherence to these standards while reinforcing the critical role of harmonization for cross-study comparability and translational reliability (Théry et al., 2018; Welsh et al., 2024). Collectively, these efforts aim to reduce technical bias, strengthen biological interpretation, and accelerate the translation of EV research into robust clinical and therapeutic applications

## Extracellular Vesicles in the Nervous System

### Cellular sources and physiological functions of extracellular vesicles in the central nervous system

In the developmental brain, EVs contribute to decisions about the fate of neural progenitor cells, neuronal differentiation, and axon guidance, while in the adult brain, they support structural remodeling and activity-dependent plasticity (Liu and Teng, 2024). In the adult CNS, virtually all major cell types of release EVs, contributing to a complex communication network (Filannino et al., 2024). EVs secreted by neurons are enriched with synaptic proteins, neurotransmitter receptors, and specific miRNAs, thereby influencing synaptic function and circuit dynamics. They are able to modulate receptor turnover, transport signaling molecules, and precisely regulate local protein synthesis at the synapse (Antoniou et al., 2023; Solana-Balaguer et al., 2023; Filannino et al., 2024). In addition to neurons, EV-mediated glia-neuron communication is crucial for maintaining homeostasis. Astrocytes release EVs containing neurotrophic factors, metabolic substrates such as lactate and cholesterol, antioxidant enzymes and miRNAs, which support neuronal viability and plasticity. Oligodendrocyte-derived EVs provide essential metabolic support by transferring lipids and enzymes that facilitate axonal maintenance and survival and myelination, which is critical for the correct propagation of action potentials (Frühbeis et al., 2020; Luarte et al., 2020; Krämer-Albers and Werner, 2023; Ikezu et al., 2024). In parallel, microglia, the resident immune cells of the CNS, release EVs carrying cytokines, chemokines, and immunomodulatory molecules, regulating neuroinflammation and shaping neuronal and glial responses in both physiological and pathological conditions (Catalano et al., 2022; Gabrielli et al., 2022b). Together, these mechanisms provide insight into how EVs act as versatile messengers that integrate neuronal and glial functions, ensuring the stability and adaptability of CNS networks. However, while EVs are essential for maintaining neuronal homeostasis, they can also exert detrimental effects under pathological conditions. Under normal physiological circumstances, EVs act as beneficial mediators, supporting neural development, plasticity, and tissue repair. In contrast, in disease states, their ability to transport bioactive molecules can facilitate the spread of harmful signals. In such contexts, EVs may carry toxic or dysfunctional cargo that exacerbates neuronal damage and contributes to the progression of neurodegenerative processes (Dong et al., 2023; Li et al., 2023c; Wiersema et al., 2024).

### Neuroexosomes: extracellular vesicles from neural cells

**Figure 3:**
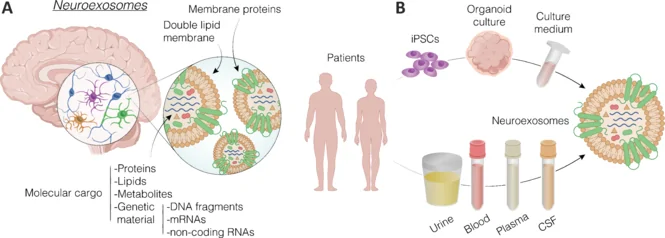
Neuroexosomes as carriers of CNS-derived molecular signatures. (A) Neuroexosomes characteristics and sources. Neuroexosomes originate from neural cells in the central nervous system and contain a diverse molecular cargo, including proteins, lipids, metabolites, and genetic material (DNA fragments, mRNA, and non-coding RNA). Their double lipid membrane is enriched with specific membrane proteins. (B) Neuroexosomes can be collected both from the conditioned medium of patient-derived induced pluripotent stem cell (iPSC) brain organoid cultures, providing an *in vitro* platform for studying disease-related pathways, and from various patient biological fluids, including urine, blood, plasma, or cerebrospinal fluid (CSF), providing minimally invasive access to central nervous system (CNS)-related molecular signatures.

Neural-derived exosomes, or neuroexosomes, do not constitute a separate category within the canonical classification of EVs. Rather, they represent a subpopulation of endosome-derived vesicles released by resident cells of the CNS, including neurons, astrocytes, oligodendrocytes, and microglia (Liu et al., 2025b). Their molecular content faithfully reflects the physiological or pathological conditions of the cells of origin, to make them valuable tools for the study of neurobiological processes. Besides cerebrospinal fluid, neuroexosomes can also be isolated from peripheral fluids, such as blood or serum, providing a direct and minimally invasive window into brain-derived material (Jiang et al., 2020; Jia et al., 2021; Herman et al., 2023; **Figure 3**). Furthermore, they can also be isolated from culture media of nerve cells (Hicks et al., 2020) or brain organoids (Pipicelli et al., 2023), offering a valuable experimental model for studying the mechanisms of biogenesis, cell communication, and other functions in a controlled environment. Strategies used for their capture and enrichment are based on the use of specific surface markers that enable the specific recovery of exosomes of neuronal or glial origin. Neuronal exosomes are usually enriched using L1 cell adhesion molecule (L1CAM), neural cell adhesion molecule 1 (NCAM), or synaptophysin, synaptotagmin, synaptosome associated protein 25 as functional markers, while astrocytic, oligodendroglial, and microglial exosomes can be identified using glial fibrillary acidic protein, myelin basic protein or myelin oligodendrocyte glycoprotein and C-X3-C motif chemokine receptor 1 or transmembrane protein 119 (TMEM119), respectively (Agliardi et al., 2023; Roseborough et al., 2023; Bravo-Miana et al., 2024; Park et al., 2024). This approach not only allows us to study the specific contributions of each cell type to the physiology and pathology of the CNS but also enables the use of neuroexosomes as promising biomarkers in neurodegenerative diseases.

## Neuroexosomes in Neurodegenerative Disorders

### Alzheimer’s disease: Extracellular vesicle–mediated transport of amyloid-β

AD is the major cause of dementia and is characterized by memory, language and cognitive dysfunction. The onset is due to the formation of amyloid-β (Aβ) plaques, which accumulate in the extracellular space of the parenchyma, and neurofibrillary tangles of hyperphosphorylated tau protein, which arise in neurons, mostly in the limbic regions and cortex (Hampel et al., 2023). Underlying this pathology lies the Aβ–tau hypothesis, which proposes that the accumulation of Aβ, occurring years before the onset of symptoms, acts as the initial trigger. This accumulation induces neuroinflammation and cellular stress, promoting a dysfunctional/pathological neuronal environment marked by cytoskeletal breakdown, synaptic disconnection, and progressive neuronal degeneration, ultimately leading to the appearance of clinical symptoms (Gabrielli et al., 2022a; Da Conceicao et al., 2025). In this scenario, EVs have been implicated in the progression of the disease, as they may contribute to the spread of AD-related pathology. Notably, both neuronal and macro- and microglial EVs contribute to this propagation, carrying pathogenic proteins and facilitating intercellular communication across different brain cell types (Falcicchia et al., 2023; Wiersema et al., 2024). In fact, these can deliver both monomers and oligomers of Aβ, promoting their extracellular accumulation, as well as hyperphosphorylated tau protein, facilitating its intra- and intercellular spread and contributing to the propagation of damage in a “prion-like” manner (Ruan et al., 2021; Gabrielli et al., 2022a). Furthermore, the spread of AD may also depend on the presence in EVs of other proteins that indirectly participate in the formation of amyloid plaques or neurofibrillary tangles through the regulation of the production, aggregation, and clearance of the Aβ peptide and tau protein.

**Figure 4:**
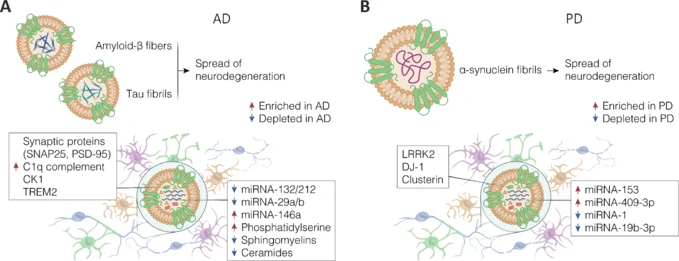
EVs drive the prion-like propagation of neurodegenerative pathology. (A, B) Neuroexosomes released by neurons and glial cells transport misfolded and hyperphosphorylated proteins, including amyloid-β, tau and α-synuclein fibrils, enabling their intercellular transmission across neural networks. This EV-mediated spreading amplifies toxic signaling, disrupts synaptic integrity, and promotes the progressive dissemination of neurodegenerative processes in AD (A) and PD (B). AD: Alzheimer’s disease; CK1: casein kinase 1; DJ-1: Parkinsonism-associated deglycase; LRRK2: leucine-rich repeat kinase 2; PD: Parkinson’s diseases; PSD-95: postsynaptic density protein 95; SNAP25: synaptosome associated protein 25; TREM2: triggering receptor expressed on myeloid cells 2.

Synaptic proteins such as synapsin, synaptotagmin, syntaxin-1, synaptosome associated protein 25 (SNAP25) and postsynaptic density protein 95 were also found in neuroexosomes, with altered profiles in AD, suggesting a role in synaptic dysfunction (Jia et al., 2021; Eitan et al., 2023). Furthermore, it has been observed that EV cargo can be enriched with complement component C1q complement, which activates the Janus kinase 2 and signal transducer and activator of transcription 1 pathway and induces beta-secretase 1 upregulation. This leads to increased β-cleavage of amyloid precursor protein (APP) within lipid rafts, resulting in excessive Aβ production and accelerating the pathological cascade of AD (Yu et al., 2025). It has been demonstrated that astrocyte-derived EVs induced by the pro-inflammatory cytokine IL-1β transport the cargo protein casein kinase 1, which promotes APP translation and the generation of Aβ in neurons (Li et al., 2023a). Furthermore, key enzymes in the metabolism of APP and fragments thereof have been identified in EVs, where they may contribute to extracellular Aβ production (Pérez-González et al., 2020). Moreover, microglial-EVs can carry triggering receptor expressed on myeloid cells 2 (TREM2) and other immune-related proteins, contributing to neuroinflammation and immune dysregulation (Gabrielli et al., 2022a; Jain and Ulrich, 2022; Zhu et al., 2025). In addition to proteins, EVs also carry a wide range of miRNAs that are able to modulate several critical cellular pathways. In physiological conditions, they play an important role in intra- and intercellular communication in order to maintain neuronal and glial homeostasis; however, in pathological contexts, oxidative stress, inflammation, and aggregated and misfolded protein accumulation alter miRNA sorting in neuroexosomes, leading to disease profiles that promote neurodegeneration (Wang et al., 2020; Ikezu et al., 2024; Wiersema et al., 2024). In AD, it has been shown that some miRNAs carried by EVs are altered and contribute to the main pathogenic mechanisms. Specifically, miR-193b and miR-34a, involved in the control of APP processing and Aβ production, appear to be significantly altered; similarly, the miR-132/212 clusters, implicated in the modulation of tau phosphorylation and the regulation of synaptic plasticity, are found to be downregulated (Belkozhayev et al., 2022; Li and Zheng, 2023; Arif et al., 2025). A further contribution arises from the miR-29 family (miR-29a/b), normally involved in limiting Aβ production, which appears to be reduced in EVs from AD subjects, promoting amyloid accumulation (Belkozhayev et al., 2022; Li and Zheng, 2023). Furthermore, EVs enriched in pro-inflammatory miRNAs, such as miR-146a, have been associated with the activation of the immune response and the amplification of neurodegenerative processes, suggesting their central role in the propagation of neuroinflammation in AD (Arif et al., 2025). EVs also carry a highly specific lipid component, which has recently been recognized as an additional pathogenic factor in AD. Lipid alteration impacts on release, content, recognition, and functional activity of EVs. Recent studies have confirmed that EVs derived from the AD-affected cerebral cortex are rich in phosphatidylserine, called vesicular “eat-me message” and other lipids that are essential components of neuronal membranes and myelin, further supporting the idea that lipid dyshomeostasis in EVs contributes to disease propagation (Su et al., 2024). Lipidomic analyses of neuroexosomes from AD patients, revealed a reduction in sphingomyelins and ceramides, which are crucial lipids for membrane stability and amyloid toxicity modulation, as well as changes in both phosphatidylcholine and phosphatidylethanolamine, affecting their release and molecular load. Changes in polyunsaturated fatty acids were also observed, with effects on inflammation and oxidative stress (Su et al., 2021). In addition, it has been shown that dysregulated levels of both cholesterol and its esters in EVs from neurons and astrocytes, as well as gangliosides, are able to modulate Aβ aggregation, while phosphatidylinositols can act on neuronal signaling pathways and cell survival (Li et al., 2023a; Krokidis et al., 2024; **Figure 4A**).

### Parkinson’s disease: Extracellular vesicle–mediated spread of α-synuclein

PD is the second most common worldwide neurodegenerative disease and is characterized by motor symptoms, such as bradykinesia, rigidity, and resting tremor, often accompanied by non-motor manifestations such as cognitive and sleep disorders (Tolosa et al., 2021). The disease is defined by the progressive degeneration of dopaminergic neurons in the substantia nigra pars compacta and the intracellular accumulation of insoluble α-synuclein aggregates, which form Lewy bodies and neurites, distributed stereotypically and progressively in multiple areas of the brain (Vázquez-Vélez and Zoghbi, 2021). Accumulation of monomeric, oligomeric, and fibrillar forms of α-synuclein and insoluble protein aggregates within neurons interferes with mitochondrial function, alters intracellular traffic dynamics, and compromises proper energy metabolism, predisposing cells to oxidative stress and vulnerability to neuronal death (Shippey et al., 2022). The activation of microglia- and reactive astrocyte-mediated neuroinflammation also plays a pivotal role in PD. These processes interplay with each other, triggering a loop that speeds up neurodegeneration and promotes the clinical progression of the disease (Yi et al., 2022; Wang et al., 2023). Similarly to AD, PD also spreads by prion-like mechanisms, contributing to synaptic dysfunction and cell death (Jan et al., 2021; Montanari et al., 2023). Originally, the association between α-synuclein and EVs occurs at the intracellular level, where its accumulation in oligomeric forms interferes with EVs trafficking by disrupting soluble N-ethylmaleimide-sensitive factor attachment protein receptors (SNARE) complex assembly (Brolin et al., 2023). However, that view is now challenged by studies that found α-synuclein in the extracellular space, specifically in human plasma and cerebrospinal fluid (Kluge et al., 2022; Herman et al., 2023; Yan et al., 2023). This indicates that α-synuclein is continuously secreted through unconventional pathways independent of the endoplasmic reticulum/Golgi system, involving interaction with the ESCRT machinery (Vandendriessche et al., 2020) and, in some cases, lipid-driven budding mediated by ceramides, thereby promoting its release in association with EVs. In addition, post-translational modifications, such as SUMOylation, a process whereby small proteins called small ubiquitin-like modifier modulate the function, stability or position of target proteins by binding to them, may act as sorting signals that further influence the packaging of α-synuclein into vesicles (Shippey et al., 2022), showing the complexity of the regulatory mechanisms governing its extracellular secretion. Besides α-synuclein, proteins such as leucine-rich repeat kinase 2 (LRRK2), Parkinsonism-associated deglycase (DJ-1), clusterin, and several apolipoproteins have been identified in EVs derived from the biological fluids of PD patients (Upadhya and Shetty, 2021). These proteins could be transported to modulate cellular function at a distance or act as a mechanism of neuronal detoxification, mirroring the cell’s attempt to export misfolded proteins or modulate the extracellular environment. This reinforces the hypothesis that EVs are not simple neurodegeneration by-products, but active carriers of pathological signals. Although most current evidence focuses on neuron-derived EVs, significant data on the involvement of glial cell-derived EVs in PD pathogenesis are emerging. It has been shown that pathological aggregation of α-synuclein can increase the secretion of astrocytic EVs (Wang et al., 2023) and, in parallel, EVs isolated from PD patients are internalized by microglia cells, leading to their activation (Vandendriessche et al., 2020). As with AD, miRNAs have also been identified in the cargo of EVs in PD. In fact, it has been shown that EVs isolated from the cerebrospinal fluid of PD patients exhibited an altered miRNA profile compared to healthy controls, with some miRNAs overexpressed (e.g., miRNA-153, miRNA-409-3p) and others reduced (e.g., miRNA-1, miRNA-19b-3p). These alterations appear to be linked to biological pathways relevant to the disease, such as neurotrophic signaling and dopaminergic synapse function (Shippey et al., 2022) (**Figure 4B**). These observations highlight EVs as potential mediators of α-synuclein propagation, neuroinflammation, and miRNA-driven dysregulation, suggesting a role in the progressive neurodegeneration characteristic of PD.

## Modeling Extracellular Vesicle–mediated Neural Communication Using Brain Organoids

**Figure 5:**
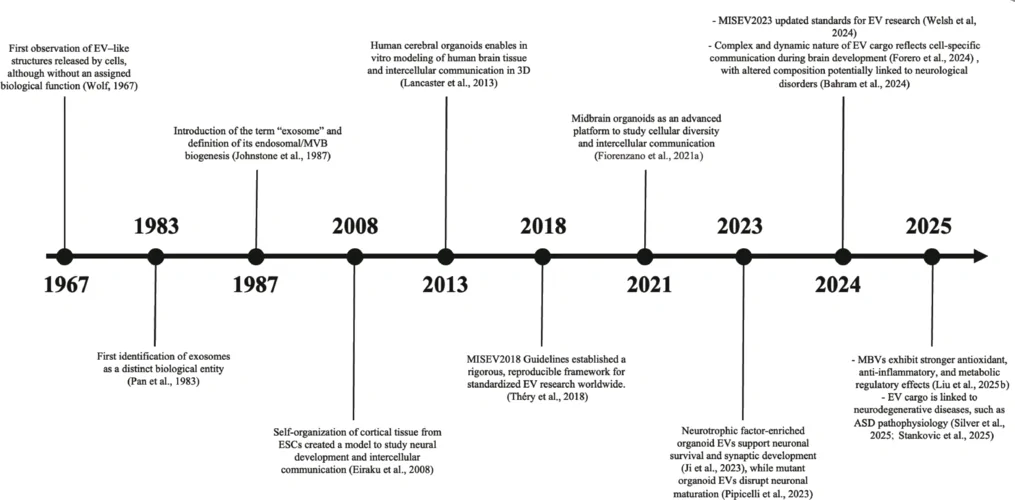
A chronological overview of the development of the EV and brain organoid fields. Key publications are highlighted to illustrate when and how these two areas converged, enabling the study of EV-mediated communication in human brain-relevant systems. ASD: Autism spectrum disorder; ESC: embryonic stem cell; EV: extracellular vesicle; MVB: multivesicular body.

EVs have emerged as key mediators of intercellular communication in the developing and diseased brain, offering both mechanistic insights and therapeutic potential. Recent studies using hPSC-derived brain organoids have advanced our understanding of EV heterogeneity, cargo specificity, and functional roles across neural populations. Liu et al. (2025a) highlight a novel EV subpopulation, matrix-bound nanovesicles (MBVs), isolated from the decellularized extracellular matrix of cerebral organoids. Compared with conventional supernatant EVs collected from culture media, organoids produce approximately ten times more MBVs. While supernatant EVs are enriched in miRNAs whose profiles evolve during organoid maturation, MBVs are characterized by higher levels of membrane-associated proteins, including integrins, and lipid species, such as glycerophospholipids and sphingolipids that support membrane rigidity and protein integration. Functionally, MBVs outperform supernatant EVs in an *in vitro* ischemic stroke model, promoting cellular recovery through enhanced autophagy, oxidative stress reduction, and anti-inflammatory effects, highlighting their therapeutic potential in neurological disorders such as ischemic stroke. Beyond MBVs, EVs derived from neural progenitors, neurons, and astrocytes within organoids exhibit pronounced cell-type-specific and developmental stage-dependent heterogeneity. Forero et al. (2024) demonstrate that neural cells selectively sort key regulators, including the mechanosensitive transcription factor YAP1, into EVs. These vesicles shuttle YAP1 to specific recipient cells, modulating cytoskeletal organization and neuroepithelial morphogenesis, thereby providing a mechanism for spatially and temporally controlled developmental signaling. Complementing these findings, Ji et al. (2023) report that organoid-derived EVs are enriched in neurotrophic factors, such as neurotrophin-4 and glial cell-derived neurotrophic factor, which enhance neuronal survival, synaptic maturation, and astroglial support. Collectively, these studies show that EV cargo composition dynamically evolves during organoid maturation, supporting the establishment of functional neural networks and precise intercellular communication. Advanced analytical technologies further illuminate the physiological roles of EVs. Using liquid chromatography–tandem mass spectrometry-based continuous organoid secretion proteomics, Yan et al. (2025) showed that EV secretion modes and cargo composition are dynamically remodeled throughout organoid development. This approach enables time-resolved tracking of secreted and vesicle-encapsulated proteins, providing a powerful framework for dissecting EV-mediated signaling and its potential dysregulation in neurological disease. The relevance of organoid-derived EVs extends to neurodevelopmental and neuropsychiatric disorders. Patient-derived organoids from individuals with Rett syndrome or autism spectrum disorder exhibit disease-specific EV alterations, including miRNAs (e.g., hsa-miR-302/367, miR-370), RNAs involved in apoptosis, metabolism, and vesicular trafficking, as well as proteins, such as cytoplasmic linker associated protein 1, which is implicated in cell polarization and axon guidance (Bahram Sangani et al., 2024; Stankovic et al., 2025). EVs derived from ventral organoids carrying mutations in LGALS3BP, associated with cortical malformations, disrupt neuronal maturation and synaptic properties in recipient cells, altering dorsoventral patterning and excitatory-inhibitory balance (Pipicelli et al., 2023). These mutant EVs modulate transcriptomic profiles of neural progenitors, demonstrating that EV-mediated signaling directly influences neuronal specification, migration, and cortical circuit assembly. EVs also reflect environmental and pathological stress responses. Organoids exposed to toxic compounds release EVs carrying molecular signatures associated with neurodegenerative disorders such as AD and PD, supporting their utility as early indicators of environmental risk (Silver et al., 2025). Similarly, EVs modulate protective mechanisms in organoid models of ischemia and spinal cord injury, including autophagy, oxidative stress response, and inflammation (**Additional Table 2** and **Figure 5**; Wolf, 1967; Pan and Johnstone, 1983; Johnstone et al., 1987; Eiraku et al., 2008; Lancaster et al., 2013; Théry et al., 2018; Fiorenzano et al., 2021c; Ji et al., 2023; Pipicelli et al., 2023; Bahram Sangani et al., 2024; Forero et al., 2024; Welsh et al., 2024; Liu et al., 2025a; Silver et al., 2025; Stankovic et al., 2025). Taken together, studies leveraging brain organoids demonstrate the dual role of EVs as mediators of normal neurodevelopment and carriers of pathological signals, establishing them as versatile tools for investigating intercellular communication, modeling neurological disorders, evaluating environmental neurotoxicity, and exploring potential EV-based therapeutic strategies. Recent advances in brain organoid platforms incorporating vascular-like networks, barrier-mimetic interfaces, and microfluidic control have substantially expanded the experimental landscape for studying EVs move, interact, and exert biological effects within structured neural environments. These systems enable investigation of EV behavior in contexts that more closely recapitulate the spatial organization, compartmentalization, and transport constraints of the human brain, including those imposed by barrier-like architectures. In parallel, the integration of organoid-on-chip technologies with biophysical and computational modeling frameworks offers new opportunities to quantitatively describe EV transport dynamics, tissue penetration, and target engagement across defined microenvironments. Such combined experimental–computational approaches can help disentangle the relative contributions of diffusion, active transport, and cellular uptake, while enabling predictive modeling of EV distribution and signaling outcomes. In this context, organoid-based models provide a powerful platform to explore how vesicle-associated signals reach specific neural targets, how their spatial distribution is shaped by tissue architecture, and how biological activity is modulated by local microenvironmental cues. Moreover, these systems offer a controlled yet physiologically relevant setting for the functional evaluation of EV-based strategies, including vesicles engineered with defined molecular payloads, allowing assessment of efficacy, selectivity, and potential off-target or adverse effects before translation into *in vivo* studies (Lancaster et al., 2013; Quadrato et al., 2017).

**Additional Table 2:** A summary of EVs in organoid models

Author	Organoid type	Key findings
Pipicelli et al. (2023)	LGALS3BP-mutant ventral cerebral organoid; assembloid	Altered dorsoventral patterning causes dorsalized cells and impaired migration
Ji et al. (2023)	Cerebral organoid	OExo enriched with NT-4 and GDNF promote neuronal survival, synaptic maturation, and astroglial support
Forero et al. (2024)	Cerebral organoids	EVs show cell, region, and developmental stage-specific composition; distinct EV uptake and transcription factor transfer (e.g., YAP1) mediate regulated cell-specific communication during brain development
Bahram Sangani et al. (2024)	MeCP2:R255X mutant forebrain organoid	Altered EV miRNA profiles (e.g. hsa-miR-302/367 cluster and C14MC miRNA species) may influence normal and RTT-affected neuronal development
Liu et al. (2025a)	Forebrain and hindbrain organoid	MBVs show higher protein and lipid content and lower miRNA expression, and stronger antioxidant, anti-inflammatory and metabolic regulatory effects than SuEVs
Silver et al. (2025)	Cerebral organoid	Cerebral-organoid-derived EV miRNAs are associated with neurodegenerative diseases, neurological pathways and may model EV-mediated neurotoxicity causing neuronal damage
Stankovic et al. (2025)	Patient ASD-derived cortical organoid	Dysregulated ASD-EVs may contribute to ASD pathophysiology via disrupted neuronal connectivity and synaptic function
Yan et al. (2025)	Cerebral organoid	COSEP-LCM revealed dynamic remodeling of EV secretion modes and cargo composition during organoid development

The table lists, for each study, the first author, the organoid subtype examined, and the main findings, including changes in EV release, cargo composition (proteins, miRNAs, and lipids), and associated functional effects on intercellular signaling or disease modelling. ASD: Autism spectrum disorder; C14MC: chromosome 14 microRNA cluster; COSEP-LCM: continuous organoid secretion/encapsulation proteome liquid chromatography-tandem mass spectrometry; EV: extracellular vesicle; LGALS3BP: galectin 3 binding protein gene; GDNF: glial cell line-derived neurotrophic factor; MBV: matrix-bound nanovesicle; MeCP2: methyl-CpG-binding protein 2; miRNA: microRNA; NT-4: neurotrophin-4; OExo: exosomes from cerebral organoids; RTT: Rett syndrome; SuEV: small urinary extracellular vesicle; YAP: Yes-associated protein 1.

## Limitation, Challenges, and Future Perspectives

EVs are central to the spreading of disease-associated signals in neurodegenerative disorders, but distinguishing causality from correlation remains challenging. In multifactorial diseases, such asAD and PD, EV release reflects pathogenic redistribution, cellular stress responses, and sometimes adaptive or protective mechanisms. Disease-associated cargo should therefore be treated as hypothesis-generating, requiring functional validation to establish direct roles in disease.

Brain organoids provide a human but relatively controlled environment to study EVs, overcoming limitations of animal models and complex tissues. Understanding EV-mediated communication during development is also critical for guiding the differentiation of pluripotent stem cells into functional, translationally relevant cell types, improving organoid fidelity and regenerative applications.

Despite these advantages, significant technical challenges remain in the isolation and characterization of EVs from organoid systems. These include contamination from extracellular matrix components (e.g., Matrigel or other hydrogels), co-isolation of serum-derived EVs from culture supplements, interference from lipoproteins and protein aggregates, and pronounced batch-to-batch variability in organoid differentiation and EV yield. These drawbacks can potentially affect EV purity, molecular profiling, and data interpretation, showing the need for carefully optimized experimental workflows and appropriate controls. Accurately assigning EVs to specific cell types remains challenging, but integrative strategies, genetic or reporter labeling, functional assays, and multi-omic profiling, can strengthen conclusions. Adopting standardized and transparent experimental practices will improve reproducibility, facilitate cross-study comparisons, and accelerate the use of organoid-derived EVs for mechanistic studies, biomarker discovery, and therapeutic development.

Future perspectives also point to the growing potential of EVs as drug delivery vehicles. Due to their ability to encapsulate and protect bioactive molecules, EVs can be engineered to deliver therapeutics, such as small molecules, RNA, or proteins, to specific target cells, potentially reducing off-target effects and improving treatment precision. This innovative application could significantly enhance drug delivery strategies in neurodegenerative diseases, where targeted therapies are urgently needed (Lian et al., 2022; Kumar et al., 2024).

In this scenario, community-driven initiatives, such as EV-TRACK, offer an important resource by promoting transparency in the reporting of experimental workflows, quality controls, and analytical parameters. Systematic sharing of methodological information through publicly accessible platforms facilitates comparison between studies, improves reproducibility, and supports the maturation of the field, particularly for emerging and technically complex models such as brain organoids and organoid-derived EVs (EV-TRACK Consortium et al., 2017).

## Additional files:

***Additional Table 1:*** *An overview of extracellular vesicle subtypes.*

***Additional Table 2:*** *A summary of EVs in organoid models.*

## Data Availability

*All data generated or analyzed in this work are included in this published article and its Additional files.*
